# Hospitals and Insured Patients

**Published:** 1913-02-01

**Authors:** Robert G. E. Whitney

**Affiliations:** Assistant Secretary. The National Hospital for Diseases of the Heart, 32 Soho Square, London, W.


*To the Editor of* The Hospital.

### Hospitals and Insured Patients.

*To the Editor of* The Hospital.

Sir,—I am desired to inform you that at a meeting of the Committee of Management of this hospital, held on January 16, 1913, the following resolution was passed: "That no change be made in the practice of this hospital in the treatment of patients in consequence of the National Health Insurance Act, but that a list of insured patients and the societies to which they belong be kept."

I should inform you that if you should desire to publish this in your paper, along with the other resolutions that have been published, my Committee will be very pleased.—Yours very truly,

Robert G. E. Whitney,
Assistant Secretary.

The National Hospital for Diseases of the Heart,
32 Soho Square, London, W., January 23.

